# Effect of Bio-Herbicide Application on Durum Wheat Quality: From Grain to Bread Passing through Wholemeal Flour

**DOI:** 10.3390/plants13202859

**Published:** 2024-10-12

**Authors:** Umberto Anastasi, Alfio Spina, Paolo Guarnaccia, Michele Canale, Rosalia Sanfilippo, Silvia Zingale, Giorgio Spina, Andrea Comparato, Alessandra Carrubba

**Affiliations:** 1 Di3A—Department of Agriculture, Food and Environment, University of Catania, Via Santa Sofia 98, 95123 Catania, Italy; umberto.anastasi@unict.it (U.A.); silvia.zingale@phd.unict.it (S.Z.); giorgiospina80@gmail.com (G.S.); 2 Council for Agricultural Research and Economics (CREA), Research Centre for Cereal and Industrial Crops, Corso Savoia, 190, 95024 Acireale, Italy; michele.canale@crea.gov.it (M.C.); rosalia.sanfilippo@crea.gov.it (R.S.); 3 DSAAF—Department of Agriculture, Food and Forest Sciences, University of Palermo, Viale delle Scienze, 4, 90128 Palermo, Italy; andreacomparato88@gmail.com (A.C.); alessandra.carrubba@unipa.it (A.C.)

**Keywords:** breadmaking, grain characteristics, bioherbicidal treatment, plant aqueous extracts, quality, *Triticum turgidum* ssp. *durum*

## Abstract

Using plant extracts to replace traditional chemical herbicides plays an essential role in sustainable agriculture. The present work evaluated the quality of durum wheat cv Valbelice in two years (2014 and 2016) using plant aqueous extracts of sumac (*Rhus coriaria* L.) and mugwort (*Artemisia arborescens* L.) as bio-herbicides on the main quality characteristics of durum wheat. The untreated, water-treated, and chemically treated durum wheat products were also analyzed as controls. Following the official methodologies, grain commercial analyses and defects of the kernels were determined. The main chemical and technological features were determined on the wholemeal flour: proteins, dry matter, dry gluten, gluten index, colorimetric parameters, mixograph, falling number, and sedimentation test in SDS. An experimental bread-making test was performed, and the main parameters were detected on the breads: bread volume, weight, moisture, porosity, hardness, and colorimetric parameters on crumb and crust. Within the two years, grain commercial analyses of the total five treatments showed no statistically significant differences concerning test weight (range 75.47–84.33 kg/hL) and thousand kernel weight (range 26.58–35.36 kg/hL). Differently, significant differences were observed in terms of kernel defects, particularly starchy kernels, black pointed kernels, and shrunken kernels, mainly due to the year factor. Analyses on the whole-grain flours showed significant differences. This affected dry gluten content (7.35% to 16.40%) and gluten quality (gluten index from 6.44 to 45.81). Mixograph results for mixing time ranged from 1.90 min to 3.15 min, whilst a peak dough ranged from 6.83 mm to 9.85 mm, showing, in both cases, statistically significant differences between treatments. The falling number showed lower values during the first year (on average 305 s) and then increased in the second year (on average 407 s). The sedimentation test showed no statistically significant differences, ranging from 27.75 mm to 34.00 mm. Regarding the bread produced, statistically significant year-related differences were observed for the parameters loaf volume during the first year (on average 298.75 cm^3^) and then increased in the second year (on average 417.33 cm^3^). Weight range 136.85 g to 145.18 g and moisture range 32.50 g/100 g to 39.51 g/100 g. Hardness range 8.65 N to 12.75 N and porosity (range 5.00 to 8.00) were closely related to the type of treatment. Finally, the color of flour and bread appeared to be not statistically significantly affected by treatment type. From a perspective of environmental and economic sustainability, the use of plant extracts with a bio-herbicidal function could replace traditional chemical herbicides.

## 1. Introduction

Knowledge of the genotypes, their selection, and genetic improvement appear to be the basis, along with knowledge of the territory, for producing better raw materials [1]. Alongside genetic improvement, there is a more outstanding commitment to environmental defense and protection that guides producers toward practices defined as greener, with farmers themselves using increasingly sustainable inputs [2,3]. The EU, through Reg. 834/2007 [4], precisely sets the rules for organic agricultural production, indicating that allowed products can be either inorganic of natural origin or organic ones [5]. Sustainable agronomic practices involve the use of plant extracts for weed control. These are useful to avoid the high persistence of chemical herbicides in the environment, with their consequent accumulation and presence in the food chain [6], as well as the development of highly resistant weed populations under organic farming, for which the use of herbicides is banned, with the specification that the latter must first and foremost be safe and effective [7]. Cereal crops require agronomic practices for weed management that generally cause quantitative losses due to lower yields and qualitative defects [8].

Increased consumer awareness of the risks involved in consuming and using food from conventional or integrated agriculture is another element that is increasingly pushing farmers toward the use of natural and sustainable products.

It has been seen, for example, how chemically treated foods retain phytosanitary residues not only in the fresh product but also after industrial processing.

Durum wheat plays a vital role in the consumer’s diet. Its use ranges from pasta to bread and, especially in some areas of the world, to a large number of other excellent food products (cous-cous, bulghur, pizza, etc.), achieving considerable importance in the Mediterranean basin and in other parts of the world (Canada, Jordan, Tunisia, Morocco, etc.), where cultivation is widespread and whose first processing product (semolina) is used not only to obtain pasta, couscous, bulgur, and other local foods but also to produce bread. In Italy, particularly in the southern regions and on the two largest islands, durum wheat is widely used in bread making. The high social, economic, and food importance of wheat (hard and soft) means that farmers engage in cultivation strategies to ensure healthy products, also including organic farming. 

However, a fairly obvious limitation related to the adoption of organic choices is due to the presence of weeds that compete for nutrients, water, and light with the wheat crop, causing yield losses, sometimes high, compared to conventional cultivation. In addition, in organic wheat fields, due to the high competition between plants, there is a greater spread of fungal diseases that attack wheat. In fact, fungal diseases such as “rusts” and “eyespot” of wheat usually have a higher incidence in organic wheat fields and are even more accentuated by the climate changes that have been occurring in recent years. 

Faced with this scenario, the cereal farmer has to choose between conventional production, capable of ensuring satisfactory yields, and organic production, which is of interest to the market and safe to the consumer but often allows a lower productivity.

For this purpose, durum wheat farmers need to improve yield performances and quality characteristics to offer a product in line with consumer expectations [9]. To this end, the adoption of cropping systems aimed at minimizing chemical residues on crops and ensuring satisfactory yields for an increasing market is stimulating researchers to investigate the use of natural substances with both herbicide and fungicide functions.

These quality aspects come through weed control, which, as mentioned, must aim at a market increasingly oriented towards organic products. A market that requires a change in traditional agronomic practices, promoting new ones that affect both the quality of the cereals and the economic profitability of the farms [10]. To date, there is very limited work on the use of plant extracts on wheat for herbicidal purposes, and straightforward studies on the quality characteristics of grain, semolina, and breads are almost absent. Therefore, this study could be a starting point and give some interesting information. Starting from the definition of the commercial features of durum wheat kernels, the present study aims to evaluate the quality of durum wheat cv Valbelice in two years (2014 and 2016) under five different agronomic conditions: treatment with plant extracts from sumac (*Rhus coriaria* L.) and mugwort (*Artemisia arborescens* L.), no treatment, and chemical treatment, treatment with only water. 

## 2. Results and Discussion

Results on the grain commercial features and defects in the two years are shown in Table 1. The data show a significant difference between the samples due to the year effect (Table 2), except for the thousand kernel weight. Significant variability was assessed also for the type of treatment (Table 3) except for test weight and thousand kernel weight.

The climatic trend of the two test years was markedly different [11], with overall better conditions in 2014 than in 2016. Indeed, in the first trial year, a higher rainfall amount was recorded throughout the crop cycle (about 390 mm vs. 209 from December to June, in both years, respectively). This different trend can be claimed to be responsible for some defects recorded in the samples from the second trial year, which significantly influenced the quality parameters of both the grains and the wholemeal flours. Concerning test weight, despite some degree of variability between the first and second year, no significant differences were detected, with a range from 75.47 (*A. arborescens*-16) and 84.33 kg/hL (Untreated-14). Considerable variability was assessed for the thousand kernel weight, which tended to increase from 2014 to 2016 (26.58 g and 35.36 g, respectively), mainly due to the climatic trend (Table 1).

The quality traits related to kernel weight and filling are strongly influenced by environmental stresses (water shortage, drought, high temperatures), which then result in the typical defects and also affect the characteristics related to test weight and thousand kernel weights, which are low on average.

For starchy kernels, the samples showed significant variability between the two years, ranging from 27.50% in Water-14 to 0.00% in the 2016 samples (excluding *R. coriaria*-16, with 1.50%) (Table 1).

Analyzing this issue, in agreement with other authors [12] discussing wheat quality, in the present work a correlation was found between the incidence of black pointed kernels and the weight of the kernels, which tends slightly to increase with the greater presence of this defect.

Lastly, shrunken kernel defects increased from the first to the second year (Table 1), with higher results in both years in *A. arborescens*, which increased from 12% in 2014 to about 40% in 2016.

The increase in starchy composition, due to the starchy kernel defect, and the scarce development of kernels due to internal and external characteristics, causing shrunken kernels, should be evaluated appropriately as they negatively affect grain yield and, therefore, semolina yield [13].

Major defects in commercial features may not only be associated with the different types of treatment but also with environmental stress affecting their development. Indeed, even though the protein quantity is optimal, the incidence of shrunken kernels is a serious defect and must also be evaluated based on the water availability of the year. The same conclusion was reached by Guzman et al. [14], who found strong drought stress and strong heat stress significantly affecting grain size, both for test weight and thousand kernel weight, with strong heat stress having the most negative influence, with average reductions of 5 percent and 27 percent, respectively.

As reported in Table 2, protein content ranged between 11.60 (g/100 g d.m.) in Chemical-14 and 18.23 (g/100 g d.m.) in A. arborescens-16, values slightly higher than those found by other authors on cv. Valbelice [15]. This higher protein content could be related to the type of semolina used, which was whole grain milled so that the total amount of protein also adds up to that of the bran components, which, although at a lesser extent, make their contribution to the total amount [16]. 

Dry matter content fluctuates for all samples around 88% in the first year and 91% in the second year. These moisture values are in line with Italian law No. 580/67 [17] on quality parameters for bread and durum wheat flour and its amendments and additions [18].

Dry gluten ranged between 7.35% in Chemical-14 and 16.4% in *A. arborescens*-16, whilst the gluten index had a range of variation between 6.44 in Untreated-14 and 45.81 in *A. arborescens*-16, presenting values for the second year in line with what reported by other authors on similar Mediterranean crops [19].

The data reported in Table 2 show a significant difference between the samples due to the year effect. In contrast, no significant variability was assessed regarding the type of treatment except for protein content, which was higher in *R. coriaria* L. and *A. arborescens* L. in both years.

Table 3 shows the colorimetric data of whole-wheat flour obtained from the different durum wheat samples. Brown index values ranged from 10.66 in *R. coriaria*-14 to 19.92 in Water-16. As expected for whole products, such values were naturally higher than those found by other authors on semolina [20,21].

Regarding the red and yellow indexes, the highest values were related to the samples from the Water-16 treatment: 2.05 (a*) and 18.30 (b*), respectively. In contrast, the lowest values were found in Chemical-14 for a* (0.41) and in Water-14 for b* (15.07). No statistically significant differences showed up according to the different types of treatment (Table 3), whereas a strong effect was evidenced according to the cultivation year (Table 3). 

The mixing time data (Table 4) show some statistical variability, ranging from lower values in Untreated-14 (1.90 min) to higher values in *R. coriaria*-16 (3.15 min). At the same time, for peak dough, there is a slightly smaller statistical difference that would suggest a non-influence related to the different treatments, ranging from 6.83 mm in Chemical-16 to 9.85 mm in *R. coriaria*-14, confirming what was previously found by Carrubba et al. [22].

Falling number values show low amylase activity in the second-year samples compared to optimal values between 200–250 s [23,24], with an extensive range varying from 65.50 s in *R. coriaria*-14 to 438.00 s in *R. coriaria*-16 samples.

The sedimentation test in sodium dodecyl sulfate (SDS) allows the qualitative/quantitative aspects of the flour to be evaluated regarding protein content and its suitability for breadmaking [25]. This trait was not significantly dependent either by treatment type or by years (Table 4), ranging from 27.75 mm (*A. arborescens*-14) to 34.0 mm (Chemical-14).

**Figure 1:**
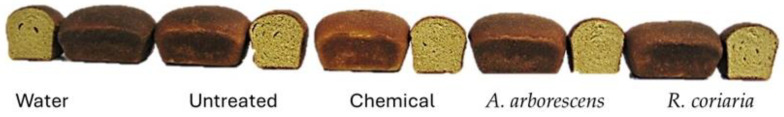
Experimental groups of bread loaves baked using flour from grains of wheat submitted to the different treatments: water-treated, untreated, chemically treated, treated with plant aqueous extracts of mugwort (*Artemisia arborescens* L.) and of sumac (*Rhus coriaria* L.) (year of research 2016).

The main results from the baking test (Figure 1), as presented in Table 5, show statistically significant differences among the treatments. The Untreated-16 sample exhibited the highest volume (430.13 cm^3)^, whilst *R. coriaria*-14 showed the lowest, with 270.00 cm^3^. The major height was observed in *A. arborescens*-16 (69.27 mm), whilst the lowest one was recorded in Water-14 (40.50 mm).

The loaf weight averaged around 144 g in first-year samples and about 139 g in second-year samples. The type of treatment was not statistically significant. Moisture content ranged from 33 (g/100 g) to 38 (g/100 g), showing a significant difference between years but not for the treatment type (Table 5).

Hardness was not statistically significant for all sources of variability. Crumb porosity was highly and irregularly developed only in the bread produced with flours from the chemical treatment (5.75), whilst in all other treatments, it appeared regular and developed from moderately (values 6.25 to 6.50) to poorly (value = 7.50).

Regarding the colorimetric parameters of the bread (Table 6), the brown index values of the crumb ranged from 45.33 in Water-14 to 32.41 in Chemical-16, whilst for the crust, the brown index was high and varied between 60.26 in Chemical-16 and 65.51 in Water-16.

Red index values showed high statistical variability in the crumb for the different treatments and the two crop years (Table 6), ranging between 4.54 in *R. coriaria*-14 and 2.44 in Untreated-16, whilst they were not significantly different for the crust, ranging between 7.91 in Water-16 and 12.39 in Untreated-14.

The b* values appeared higher and had a little variable in the crumb, which defines a more “yellow” post-cooking appearance (23.73 in Water-14 and 27.11 in *R. coriaria*-16), compared to the crust, where no statistical difference was noted among the samples (from 12.45 in Water-16 to 18.25 in Chemical-14).

### Principal Component Analysis

**Figure 2:**
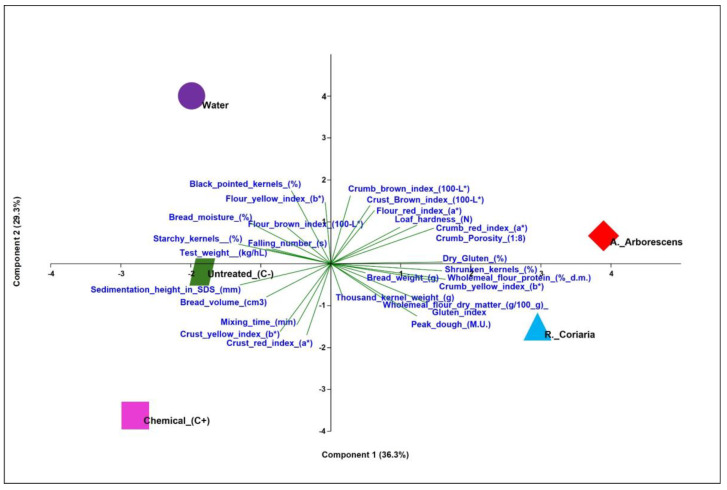
Principal component analysis (PCA) biplot, defined by the first two principal components. Vectors represent the loadings of the physical, chemical, and technological quality characteristics of grain, flours, doughs, and breads obtained in 2014 and 2016 from five treatments, including plant extracts from *Rhus coriaria* L. and *Artemisia arborescens* L., no treatment, chemical treatment, and treatment with only water.

The principal component analysis revealed that the first two components accounted for 65.6% of the total explained variance (PC1 = 36.3%; PC2 = 29.3%) (Table S1). The treatments with plant extracts were clearly distinguished from the controls in the multidimensional space (Figure 2). Wholemeal flour protein (% d.m.), dry gluten (%), shrunken kernels (%), crumb yellow index (b*), crumb porosity (1:8), gluten index, crumb red index (a*), and peak dough (M.U.) showed a strong positive correlation (>72.0%) with PC1 (Table S2). The two plant extracts, both positioned in the positive portion of PC1, were characterized by high values for these parameters. In contrast, starchy kernels (%), sedimentation height in SDS (mm), and bread moisture (%) showed a strong negative correlation, representing the three controls, positioned in the negative section of PC1 (Table S2, Figure 2). Black pointed kernels (%), crumb brown index (100-L*), flour yellow index (b*), crust brown index (100-L*), and flour red index (a*) showed a strong positive correlation with PC2, which represented the water-only control, positioned in the positive portion of PC2. Additionally, a strong negative correlation with PC2 was noted for crust red index (a*), crust yellow index (b*), mixing time (min), and peak dough (M.U.), which primarily characterized the chemical treatment, positioned in the negative portion of PC2 (Table S2, Figure 2).

## 3. Materials and Methods

### 3.1. Preparation and Use of Plant Water Extracts

Plant extracts used for the tests were obtained from both the leaves and inflorescences of wild plants of *Rhus coriaria* L. and *Artemisia arborescens* L. collected in both years before field treatments near Ciminna (Palermo, Sicily) and Sparacia (Cammarata, Agrigento, Sicily), respectively. The extraction method described by Militello & Carrubba [26] was followed.

### 3.2. Field Management

The field trials were carried out in 2014 and 2016 in the experimental farm “Sparacia” (Cammarata, AG, Sicily; 37°38′ N 13°46′ E; 415 m a.s.l.) of the Department of Agricultural and Forest Sciences of the University of Palermo. In both years, the experiment was performed on a durum wheat field, managed according to the cropping techniques ordinarily applied in the cereal-growing areas of the site [11]. Sowings were made mechanically in mid-December, distributing on rows 30 cm apart about 200 kg ha^−1^ of seed (350 viable seeds per m^2^). Fertilization included about 70 kg ha^–1^ of P_2_O_5_ and 120 kg ha^–1^ of N, applied in part before sowing time (all P_2_O_5_ and one half of the total N amount), whereas the remaining N fertilization was distributed at the full tillering stage.

All treatments and controls were laid out according to a randomized block design with three replicates, with elementary plots of 4.54 m^2^. The chosen durum wheat variety was Valbelice (0111 × BC5). This variety was obtained in 1992 by the Department of Agricultural and Forest Sciences of the University of Palermo. It is an early, high-yielding, and tall genotype, which is generally able to compete with weeds and is strongly suitable for organic farming and low-input cropping systems [27,28]. Further details about the agronomic management, including bioherbicide treatment and harvesting, have been reported in previously published papers [22].

### 3.3. Determination of Grain Characteristics and Defects

Representative samples of grain per individual plot were used to determine thousand kernel weight (TKW), test weight (TW), starchy kernels, shrunken kernels, and black-pointed kernels. TKW was determined by weighing eight sub-samples of 100 kernels, and the average weight was relative to 1000 kernels. TW was determined with a Test Weight Module (TWM) installed under the Infratec 1241 Grain Analyser (Foss Tecator, Höganas, Sweden).

Starchy kernels, shrunken kernels, and black-pointed kernel percentages were visually estimated on representative sub-samples of kernels (30 g).

Kernels were sorted visually into wholly vitreous grains and not (at least two spots), and the latter count was expressed as a percentage of the total kernels. Worldwide recognized procedures define fully vitreous kernels as ‘those that do not disclose the slightest trace of farinaceous endosperm’ [13]. All analyses were performed in triplicate.

### 3.4. Grain Milling

The grain from the three field replicates of each year was ground using an experimental mill to obtain wholemeal flour (Cyclotec type 120, Falling Number, Huddinge, Sweden) with a 0.5-mm sieve [22].

### 3.5. Physico-Chemical Analyses of Wholemeal Flour 

The protein and moisture content of grain samples were determined using the Infratec 1241 grain analyzer (Foss Tecator, Hoganas, Sweden).

The gluten quantity (wet and dry gluten content) and quality (gluten index) were evaluated using a Glutomatic 2200 apparatus, a Centrifuge 2015, and a Glutork 2020 (Perten Instruments AB, Huddinge, Sweden) according to the ICC Standard No. 158 [29]. Centrifugation was performed to force the wet gluten through a specially constructed sieve under standardized conditions. Wet gluten passing through the grid is called the “B fraction”; when this is highly represented, it indicates the poor technological quality of the gluten according to the AACC 38-12 method [30].

The CR 200 Minolta Colorimeter Chroma (Minolta, Osaka, Japan) was used to evaluate the colorimetric parameters of both whole-wheat flours and breads.

Colorimetric measurements on wholemeal flours were performed following the CIELab colorimetric model [31], to express the results through the following indices: L*, a* (red index), and b* (yellow index).

All analyses were performed in triplicate.

### 3.6. Technological Tests on Wholemeal Flour Dough

Mixographic analysis allows the analysis of small amounts of flour to measure dough strength. The mixographic curve was obtained using the National MFG. Mixograph. Co. (Lincoln, NE, USA), following the AACC 54-40.02 method [30].

The α-amylase activity was determined using the Falling Number 1500 apparatus (Perten Instruments AB, Huddinge, Sweden), following the method ISO 3093 [32].

The sodium dodecyl sulfate (SDS) sedimentation test (Sigma-Aldrich, Milan, Italy) is a useful preliminary test for estimating gluten quality. This test was performed according to the method of Dick and Quick [33].

All analyses were performed in triplicate.

### 3.7. Baking Test

The breads were obtained according to the official AACC 10-10.03 methodology [30], described by Sanfilippo et al. [34].

Physical characteristics such as volume, height, weight, moisture, hardness, crumb porosity, crumb color, and crust color were evaluated on the resulting bread.

The volume was determined based on the displacement of rape seeds in a loaf volume meter according to the AACC 10-05 method [30]; loaf height was measured using a digital calliper (Digi-MaxTM, Scienceware^®^, Wayne, NJ, USA). Weight was measured using a digital scale (OHAUS mod. Adventurer pro AV2102C, Pine Brook, NJ, USA).

The loaf hardness was measured using a texture analyzer (Zwick Z 0.5 Roell, Ulm, Germany) equipped with an aluminum 8-mm diameter cylindrical probe.

For porosity assessment, the central bread slices of each loaf were taken and visually compared with the eight Dallmann reference images, representing the cross-section of loaves with different crumb structures. Crumb porosity was evaluated based on the 8-grade Mohs scale as modified by Dallmann (1981) [35], where 1 indicates non-uniform structure, large and irregular cells, and 8 indicates uniform compact structure, small and regular cells [36].

Moisture was measured according to the method described in Section 3.2, while the color of both crust and crumb was evaluated according to the procedure described in Section 3.3.

All analyses were performed in triplicate.

### 3.8. Statistical Analysis

Results are reported as mean ± standard deviation. All statistical analyses were performed using the Statgraphics^®^ Centurion XVI software package (https://www.statgraphics.com) (Statpoint Technologies, Inc., The Plains, VA, USA). One-factor and two-factor analyses of variance (ANOVA), followed by Tukey’s HSD test (*p* < 0.05, *p* < 0.01, *p* < 0.001), were carried out on all grain characteristics, chemical, colorimetric, technological, and breadmaking attributes.

The following two factors were considered: 1. the year of field trials; 2. type of treatment. A one-factor analysis determined the interaction of the factors studied, while a two-factor analysis analyzed each factor’s influence (or lack of influence) individually. 

A principal component analysis (PCA) was performed on the dataset containing the physical, chemical, and technological quality characteristics of grain, flours, doughs, and breads obtained in 2014 and 2016 from five treatments, including plant extracts from *Rhus coriaria* L. and *Artemisia arborescens* L., no treatment, chemical treatment, and treatment with only water. PCA was conducted using the Paleontological Statistics (PAST) software package, 2011 [37].

## 4. Conclusions

The use of plant extracts in weed control remains of primary importance in a sustainable agriculture context, especially for greater eco-sustainability of agronomic practices.

From a perspective of environmental and economic sustainability, the use of plant extracts with a bio-herbicidal function could replace traditional chemical herbicides.

The importance of the use of plant extracts to replace traditional chemical herbicides is evident both from an environmental perspective and with regard to the health of agricultural operators and consumers. The latter aspect is very much felt, especially by the modern consumer, who is increasingly attentive to the “naturality” of products and their possible health benefits.

The results obtained showed that the use of natural herbicides can have almost the same herbicidal effect as chemical herbicides, both from a commercial point of view (commodity characteristics of the grain) and from a chemical-physical and technological point of view (semolina, doughs, and breads).

Commercial quality parameters such as thousand-seed weight and hectoliter weight showed no treatment-related differences in either year. The observations on the quality of semolina and breads enlightened statistical differences related to years rather than treatments.

It appears that the use of plant extracts for weed control does not cause any quality depreciation of the kernels and semolina, nor does it bring about a deterioration in the chemical-physical and technological characteristics of the doughs and breads derived from them. Breads obtained from the two treatments with extracts of *R. coriaria* and *A. arborescens* showed similar quality characteristics to those recorded from the untreated, chemically and water-treated samples.

The Principal Component Analysis revealed that the first two components accounted for 65.6% of the total explained variance (PC1 = 36.3%; PC2 = 29.3%) (Table S1). The treatments with plant extracts were clearly distinguished from the controls in the multidimensional space.

Although preliminary, the results so far have shown that the use of these natural substances can be useful from the perspective of environmental sustainability and circular economy.

The topic is new and deserves further investigation. Additional studies are needed, exploring a wider range of combinations between crops and bio-herbicides.

## Figures and Tables

**Table 1:** Factorial ANOVA of main grain characteristics and defects obtained in 2014 and 2016 from the five treatments (data are means ± standard deviations).

Factors of Variability		Test Weight (kg/hL)	Thousand Kernel Weight (g)	Starchy Kernels (%)	Black Pointed Kernels (%)	Shrunken Kernels (%)
Year (A)	2014	82.66 ± 2.50 a	30.08 ± 5.40	17.40 ± 9.13 a	7.80 ± 3.90 a	5.20 ± 4.21 b
	2016	77.20 ± 2.99 b	33.45 ± 1.48	0.30 ± 0.67 b	0.20 ± 0.26 b	34.75 ± 4.24 a
Treatment type (B)	Water	81.34 ± 3.33	30.38 ± 5.80	13.75 ± 15.90 a	6.63 ± 7.54 a	16.25 ± 16.52 b
	Untreated (C−)	80.18 ± 5.68	32.71 ± 0.48	7.50 ± 8.81 ab	5.00 ± 5.83 ab	19.00 ± 19.33 ab
	Chemical (C+)	79.86 ± 5.24	31.28 ± 5.06	13.00 ± 15.21 a	2.25 ± 2.60 b	17.13 ± 16.95 b
	*A. arborescens*	77.44 ± 3.07	29.28 ± 4.90	7.00 ± 8.12 ab	3.88 ± 4.66 ab	25.88 ± 16.14 a
	*R. coriaria*	80.84 ± 1.70	35.18 ± 1.35	3.00 ± 1.79 b	2.25 ± 2.02 b	21.63 ± 16.95 ab
A × B	Water-14	84.20 ± 0.57 a	27.15 ± 7.52	27.50 ± 1.41 a	13.00 ± 2.83 a	2.00 ± 1.41 b
	Untreated-14	84.33 ± 0.53 a	32.71 ± 0.59	15.00 ± 2.83 bc	10.00 ± 1.41 ab	2.50 ± 0.71 b
	Chemical-14	84.25 ± 1.77 a	28.96 ± 7.39	26.00 ± 4.24 ab	4.50 ± 0.28 ab	2.50 ± 0.71 b
	*A. arborescens*-14	78.40 ± 0.57 a	26.58 ± 6.43	14.00 ± 1.41 bc	7.50 ± 3.54 ab	12.00 ± 2.83 b
	*R. coriaria*-14	82.13 ± 0.04 a	35.00 ± 1.87	4.50 ± 0.28 cd	4.00 ± 0.00 ab	7.00 ± 0.00 b
	Water-16	78.48 ± 0.46 a	33.62 ± 1.62	0.00 ± 0.00 d	0.25 ± 0.35 b	30.50 ± 2.12 a
	Untreated-16	76.03 ± 5.27 a	32.71 ± 0.59	0.00 ± 0.00 d	0.00 ± 0.00 b	35.50 ± 5.66 a
	Chemical-16	75.47 ± 1.44 a	33.61 ± 0.81	0.00 ± 0.00 d	0.00 ± 0.00 b	31.75 ± 2.47 a
	*A. arborescens*-16	76.49 ± 4.93 a	31.98 ± 1.20	0.00 ± 0.00 d	0.25 ± 0.35 b	39.75 ± 1.77 a
	*R. coriaria*-16	79.55 ± 1.41 a	35.36 ± 1.35	1.50 ± 0.71 d	0.50 ± 0.00 b	36.25 ± 2.47 a

Different letters in each column indicate a significant difference at *p* < 0.001; test weight (Sample) *p* < 0.01; *p* < 0.05 (Tukey’s test).

**Table 2:** Factorial ANOVA of main chemical parameters of the wholemeal flours obtained in 2014 and 2016 from the five treatments (data are means ± standard deviations).

Factors of Variability		Protein Content (% d.m.)	Dry Matter (g/100 g)	Dry Gluten (% d.m.)	Gluten Index
Year (A)	2014	13.32 ± 1.62 b	88.02 ± 0.29 b	8.75 ± 1.15 b	8.99 ± 2.12 b
	2016	17.01 ± 0.93 a	90.69 ± 0.11 a	14.52 ± 1.42 a	40.29 ± 5.33 a
Treatment type (B)	Water	14.05 ± 1.90 b	89.27 ± 1.52	10.39 ± 2.98	20.28 ± 14.60 b
	Untreated (C−)	14.54 ± 2.64 b	89.14 ± 1.72	11.18 ± 4.00	24.01 ± 20.48 ab
	Chemical (C+)	14.54 ± 3.42 b	89.49 ± 1.49	11.13 ± 5.34	24.33 ± 18.08 ab
	*A. arborescens*	16.74 ± 1.75 a	89.51 ± 1.53	12.93 ± 4.91	28.37 ± 20.19 a
	*R. coriaria*	15.96 ± 1.01 a	89.37 ± 1.49	12.57 ± 3.16	26.24 ± 17.79 ab
A × B	Water-14	12.40 ± 0.00 d	87.95 ± 0.07 b	8.28 ± 0.50 b	7.68 ± 1.07 b
	Untreated-14	12.25 ± 0.07 d	87.65 ± 0.21 b	8.35 ± 0.17 b	6.44 ± 1.00 b
	Chemical-14	11.60 ± 0.14 d	88.20 ± 0.14 b	7.35 ± 0.42 b	8.75 ± 0.50 b
	*A. arborescens*-14	15.25 ± 0.07 c	88.20 ± 0.28 b	9.45 ± 1.85 b	10.93 ± 0.80 b
	*R. coriaria*-14	15.10 ± 0.00 c	88.10 ± 0.42 b	10.33 ± 0.64 b	11.18 ± 1.94 b
	Water-16	15.69 ± 0.20 bc	90.58 ± 0.05 a	12.50 ± 0.04 a	32.87 ± 2.04 a
	Untreated-16	16.83 ± 0.11 abc	90.62 ± 0.04 a	14.00 ± 0.06 a	41.58 ± 4.65 a
	Chemical-16	17.48 ± 0.67 ab	90.78 ± 0.02 a	14.90 ± 0.17 a	39.91 ± 3.16 a
	*A. arborescens*-16	18.23 ± 0.53 a	90.82 ± 0.11 a	16.40 ± 0.04 a	45.81 ± 2.47 a
	*R. coriaria*-16	16.83 ± 0.25 abc	90.65 ± 0.07 a	14.80 ± 0.06 a	41.31 ± 6.10 a

Different letters in each column indicate a significant difference at *p* < 0.001 (Tukey’s test).

**Table 3:** Factorial ANOVA of colorimetric parameters of flours obtained in 2014 and 2016 from the five treatments (data are means ± standard deviations).

Factors of Variability		Brown Index (100-L*)	Red Index (a*)	Yellow Index (b*)
Year (A)	2014	16.24 ± 5.25	0.72 ± 0.42 b	15.64 ± 0.49 b
	2016	18.54 ± 1.16	1.82 ± 0.27 a	17.18 ± 0.96 a
Treatment type (B)	Water	19.63 ± 1.18	1.35 ± 0.85	16.68 ± 2.17
	Untreated (C−)	16.35 ± 2.86	1.32 ± 0.74	16.34 ± 1.10
	Chemical (C+)	18.31 ± 1.63	1.06 ± 0.76	16.30 ± 0.71
	*A. arborescens*	18.65 ± 0.90	1.54 ± 0.51	16.62 ± 0.78
	*R. coriaria*	14.01 ± 7.49	1.10 ± 0.66	16.12 ± 0.37
A × B	Water-14	19.35 ± 0.38	0.65 ± 0.39 ab	15.07 ± 0.21 b
	Untreated-14	13.90 ± 0.59	0.67 ± 0.05 ab	15.48 ± 0.21 ab
	Chemical-14	18.20 ± 2.19	0.41 ± 0.16 b	15.71 ± 0.14 ab
	*A. arborescens*-14	19.12 ± 0.81	1.13 ± 0.13 ab	16.16 ± 0.52 ab
	*R. coriaria*-14	10.66 ± 9.05	0.76 ± 0.72 ab	15.80 ± 0.07 ab
	Water-16	19.92 ± 1.90	2.05 ± 0.08 a	18.30 ± 1.92 a
	Untreated-16	18.80 ± 0.01	1.96 ± 0.03 a	17.20 ± 0.49 ab
	Chemical-16	18.43 ± 0.87	1.71 ± 0.13 ab	16.89 ± 0.27 ab
	*A. arborescens*-16	18.19 ± 0.78	1.95 ± 0.28 a	17.09 ± 0.74 ab
	*R. coriaria*-16	17.37 ± 0.53	1.44 ± 0.27 ab	16.44 ± 0.04 ab

Different letters in each column indicate a significant difference at *p* < 0.05 (sample), *p* < 0.001 (year, treatment) (Tukey’s test).

**Table 4:** Factorial ANOVA of technological quality characteristics of the flours obtained in 2014 and 2016 from the five treatments (data are means ± standard deviations).

Factors of Variability		Mixing Time (min)	Peak Dough (mm)	Falling Number (s)	Sedimentation Height in SDS (mm)
Year (A)	2014	2.29 ± 0.46 b	8.87 ± 1.20 a	305.40 ± 127.76 b	31.85 ± 3.00
	2016	2.94 ± 0.20 a	7.12 ± 0.36 b	406.95 ± 30.39 a	30.85 ± 1.40
Treatment type (B)	Water	2.52 ± 0.27	7.21 ± 0.28	363.13 ± 8.11 b	31.50 ± 1.68
	Untreated (C−)	2.50 ± 0.77	7.61 ± 0.63	412.50 ± 28.95 a	32.63 ± 0.25
	Chemical (C+)	2.88 ± 0.24	8.19 ± 1.59	374.00 ± 32.76 ab	32.25 ± 2.10
	*A. arborescens*	2.43 ± 0.57	8.53 ± 1.23	379.50 ± 26.56 ab	28.75 ± 2.53
	*R. coriaria*	2.75 ± 0.48	8.44 ± 1.90	251.75 ± 215.09 c	31.63 ± 2.90
A × B	Water-14	2.30 ± 0.14 abc	7.45 ± 0.07 abc	361.25 ± 5.40 bc	31.50 ± 2.40
	Untreated-14	1.90 ± 0.57 c	8.05 ± 0.64 abc	388.00 ± 7.00 abc	32.50 ± 0.60
	Chemical-14	2.95 ± 0.35 a	9.55 ± 0.35 ab	355.50 ± 32.40 c	34.00 ± 0.80
	*A. arborescens*-14	1.95 ± 0.07 bc	9.45 ± 0.92 abc	356.75 ± 7.80 c	27.75 ± 3.20
	*R. coriaria*-14	2.35 ± 0.21 abc	9.85 ± 1.63 a	65.50 ± 3.00 d	33.50 ± 2.40
	Water-16	2.75 ± 0.02 abc	6.98 ± 0.04 bc	365.00 ± 12.73 abc	31.50 ± 0.71
	Untreated-16	3.10 ± 0.03 a	7.18 ± 0.18 bc	437.00 ± 8.49 ab	32.75 ± 0.35
	Chemical-16	2.81 ± 0.18 abc	6.83 ± 0.18 c	392.50 ± 18.38 abc	30.50 ± 0.71
	*A. arborescens*-16	2.91 ± 0.25 ab	7.60 ± 0.49 abc	402.25 ± 1.06 abc	29.75 ± 0.35
	*R. coriaria*-16	3.15 ± 0.01 a	7.03 ± 0.39 bc	438.00 ± 4.95 a	29.75 ± 1.77

Different letters in each column indicate a significant difference at *p* < 0.01 (Tukey’s test).

**Table 5:** Factorial ANOVA of physical characteristics of bread obtained in 2014 and 2016 from flour of the five treatments (data are means ± standard deviations).

Factors of Variability		Volume (cm^3^)	Weight (g)	Moisture (g/100 g)	Hardness (N)	Crumb Porosity *
Year (A)	2014	298.75 ± 22.71 b	143.55 ± 1.46 a	33.46 ± 0.67 b	10.51 ± 1.40	6.80 ± 1.23 a
	2016	417.33 ± 8.96 a	138.73 ± 1.93 b	38.82 ± 0.56 a	10.24 ± 1.72	6.38 ± 0.57 b
Treatment type (B)	Water	346.56 ± 71.56 ab	140.73 ± 26.96	36.40 ± 3.11 a	10.25 ± 0.89	6.56 ± 0.52 b
	Untreated (C−)	376.31 ± 62.17 a	140.79 ± 27.78	36.43 ± 4.00 a	11.07 ± 1.50	6.38 ± 0.48 b
	Chemical (C+)	368.91 ± 53.66 ab	141.64 ± 25.97	36.01 ± 4.96 ab	8.89 ± 0.33	5.75 ± 0.87 c
	*A. arborescens*	354.66 ± 70.40 ab	141.98 ± 25.18	35.85 ± 3.89 b	10.90 ± 1.60	7.50 ± 0.58 a
	*R. coriaria*	343.75 ± 85.65 b	140.58 ± 24.70	36.01 ± 2.98 ab	10.77 ± 2.80	6.75 ± 1.44 b
A × B	Water-14	285.00 ± 14.14 bc	142.39 ± 0.34 ab	34.20 ± 1.20 d	10.88 ± 1.04	7.00 ± 0.00 b
	Untreated-14	322.50 ± 3.54 b	141.74 ± 1.48 ab	33.60 ± 0.50 de	10.01 ± 0.60	6.00 ± 0.00 cd
	Chemical-14	322.50 ± 3.54 b	144.15 ± 0.52 a	32.50 ± 0.20 f	9.12 ± 0.30	5.00 ± 0.00 e
	*A. arborescens*-14	293.75 ± 5.30 bc	145.18 ± 0.06 a	33.10 ± 0.20 ef	9.77 ± 0.75	8.00 ± 0.00 a
	*R. coriaria*-14	270.00 ± 7.07 c	144.32 ± 0.39 a	33.90 ± 1.10 de	12.75 ± 1.86	8.00 ± 0.00 a
	Water-16	408.13 ± 0.88 a	139.07 ± 1.41 ab	38.60 ± 0.73 bc	9.62 ± 0.34	6.13 ± 0.18 bcd
	Untreated-16	430.13 ± 0.53 a	139.84 ± 0.00 ab	39.26 ± 0.45 ab	12.13 ± 3.95	6.75 ± 0.35 bc
	Chemical-16	415.33 ± 3.08 a	139.13 ± 0.41 ab	39.51 ± 1.18 a	8.65 ± 0.71	6.50 ± 0.00 bc
	*A. arborescens*-16	415.58 ± 0.81 a	138.79 ± 0.80 ab	38.60 ± 0.23 bc	12.03 ± 0.48	7.00 ± 0.00 b
	*R. coriaria*-16	417.50 ± 14.14 a	136.85 ± 4.54 b	38.11 ± 0.34 c	8.79 ± 0.65	5.50 ± 0.00 de

* Scale 1–8; 1 = non-uniform structure, large and irregular cells; 8 = uniform compact structure, small and regular cells. Different letters in each column indicate a significant difference at *p* < 0.001; moisture, year, treatment *p* < 0.05 (Tukey’s test).

**Table 6:** Factorial ANOVA of colorimetric parameters of crumb and crust obtained in 2014 and 2016 in bread obtained from different treatments (data are means ± standard deviations).

Factors of Variability		Crumb			Crust		
		Brown Index (100-L*)	a*	b*	Brown index (100-L*)	a*	b*
Year (A)	2014	41.37 ± 2.55 a	3.92 ± 0.52 a	24.35 ± 0.54 b	63.26 ± 1.23	10.69 ± 1.22	16.08 ± 1.78
	2016	32.86 ± 0.64 b	2.35 ± 0.12 b	26.61 ± 0.45 a	63.02 ± 2.76	10.44 ± 2.24	15.27 ± 2.77
Treatment type (B)	Water	38.99 ± 7.33 a	3.25 ± 1.08 ab	25.35 ± 1.88	64.29 ± 1.90	8.68 ± 1.22	13.91 ± 2.15
	Untreated (C−)	36.19 ± 3.11 b	2.99 ± 0.65 ab	25.43 ± 1.31	63.00 ± 1.07	11.46 ± 1.13	16.21 ± 1.51
	Chemical (C+)	35.84 ± 4.03 b	2.75 ± 0.54 b	25.36 ± 1.08	61.42 ± 2.44	11.94 ± 1.84	18.14 ± 2.02
	*A. arborescens*	37.21 ± 5.28 ab	3.24 ± 1.02 ab	25.57 ± 0.89	62.94 ± 3.02	10.21 ± 2.18	15.11 ± 2.90
	*R. coriaria*	37.35 ± 5.06 ab	3.44 ± 1.28 a	25.70 ± 1.64	64.05 ± 0.87	10.53 ± 0.48	15.01 ± 0.73
A × B	Water-14	45.33 ± 0.73 a	4.18 ± 0.04 ab	23.73 ± 0.17 b	63.06 ± 0.89	9.46 ± 0.66	15.37 ± 0.33
	Untreated-14	38.80 ± 1.19 bc	3.55 ± 0.13 ab	24.31 ± 0.01 ab	62.78 ± 1.39	12.39 ± 0.30	17.36 ± 0.38
	Chemical-14	39.27 ± 1.09 bc	3.21 ± 0.21 bc	24.62 ± 1.06 ab	62.59 ± 0.02	11.51 ± 0.67	18.25 ± 0.40
	*A. arborescens*-14	41.78 ± 0.50 ab	4.11 ± 0.21 ab	24.81 ± 0.16 ab	63.82 ± 2.33	9.89 ± 0.70	14.92 ± 2.54
	*R. coriaria*-14	41.67 ± 1.10 ab	4.54 ± 0.18 a	24.29 ± 0.26 ab	64.05 ± 1.49	10.21 ± 0.09	14.50 ± 0.34
	Water-16	32.66 ± 0.52 d	2.32 ± 0.17 c	26.98 ± 0.23 a	65.51 ± 2.02	7.91 ± 1.27	12.45 ± 2.30
	Untreated-16	33.58 ± 0.57 cd	2.44 ± 0.17 c	26.55 ± 0.32 ab	63.23 ± 1.15	10.53 ± 0.50	15.06 ± 1.18
	Chemical-16	32.41 ± 0.70 d	2.29 ± 0.08 c	26.11 ± 0.45 ab	60.26 ± 3.54	12.37 ± 2.99	18.03 ± 3.47
	*A. arborescens*-16	32.65 ± 0.02 d	2.37 ± 0.12 c	26.33 ± 0.17 ab	62.06 ± 4.33	10.54 ± 3.65	15.31 ± 4.32
	*R. coriaria*-16	33.02 ± 0.95 d	2.34 ± 0.12 c	27.11 ± 0.02 a	64.05 ± 0.25	10.86 ± 0.50	15.53 ± 0.64

Different letters in each column indicate a significant difference at *p* < 0.001 (Tukey’s test).

## Data Availability

All available data are reported in the paper.

## Supplementary Materials

The following supporting information can be downloaded at: https://www.mdpi.com/article/10.3390/plants13202859/s1, Table S1. Principal Component Analysis (PCA). Eigenvalue and proportion of variance explained by each principal component. Table S2. Principal component analysis (PCA) loadings of physical, chemical, and technological quality characteristics of grain, flours, doughs, and breads obtained in 2014 and 2016 from five treatments, including plant extracts from *Rhus coriaria* L. and *Artemisia arborescens* L., no treatment, chemical treatment, and treatment with only water.
