# Supplementary material for: Effect of Bio-Herbicide Application on Durum Wheat Quality: From Grain to Bread Passing through Wholemeal Flour
Source: Plants (Basel). 2024 Oct 12;13(20):2859. doi: 10.3390/plants13202859 (PMC11511445; doi:10.3390/plants13202859)
Supplement: Supplementary file 1 [file plants-13-02859-s001.zip › plants-3210870-supplementary.pdf]

Supplemental materials

Table S1. Principal Component Analysis (PCA). Eigenvalue and proportion of variance explained by each principal component.

PC	Eigenvalue	% variance	cumulative %
1	9,79491	36,277	36,277
2	7,91105	29,3	65,577
3	6,40389	23,718	
4	2,89015	10,704	

Table S2. Principal component analysis (PCA) loadings of physical, chemical, and technological quality characteristics of grain, flours, doughs, and breads obtained in 2014 and 2016 from five treatments, including plant extracts from *Rhus coriaria* L. and *Artemisia arborescens* L., no treatment, chemical treatment, and treatment with only water.

	Axis 1	Axis 2	Axis 3	Axis 4	Axis 5
Wholemeal flour dry matter (g/100 g)	0,4414	-0,4418	0,5132	-0,5887	5,15E-14
Wholemeal flour protein (% d.m.)	0,9612	-0,1962	0,1885	0,04509	-6,41E-16
Test weight (kg/hL)	-0,5405	0,1857	-0,7855	-0,2373	1,07E-14
Thousand kernel weight (g)	0,09479	-0,4018	-0,902	0,1262	4,05E-15
Starchy kernels (%)	-0,7742	0,2456	0,4436	-0,3788	-6,68E-15
Black pointed kernels (%)	-0,3306	0,9197	0,07057	0,1998	3,49E-15
Shrunken kernels (%)	0,9299	-0,08682	0,2746	0,2287	2,72E-15
Dry Gluten (%)	0,9351	0,02199	-0,3513	0,04013	1,18E-15
Gluten index	0,8019	-0,4936	0,2615	0,2119	1,13E-15
Mixing time (min)	-0,2852	-0,7659	-0,2788	-0,5043	-7,35E-15
Peak dough (M.U.)	0,7218	-0,6594	0,179	-0,1102	-2,23E-15
Falling number (s)	-0,4954	0,1901	0,6853	0,4988	2,81E-15
Sedimentation height in SDS (mm)	-0,7601	-0,2659	-0,5771	0,1356	5,76E-15
Flour brown index (100-L*)	-0,3645	0,4558	0,7625	-0,2793	-6,72E-15
Flour red index (a*)	0,3661	0,6716	0,5373	0,3553	2,52E-15
Flour yellow index (b*)	-0,04589	0,7748	0,6221	-0,1031	4,00E-15
Bread volume (cm3)	-0,539	-0,4177	0,3282	0,6536	6,09E-15
Bread weight (g)	0,289	-0,1143	0,9199	-0,2394	-1,31E-14
Bread moisture (%)	-0,6747	0,5118	-0,353	0,3978	1,32E-14
Loaf hardness (N)	0,5778	0,4579	-0,275	0,6172	9,59E-15
Crumb Porosity (1:8)	0,8632	0,4479	0,1905	0,1341	3,39E-15
Crumb brown index (100-L*)	0,1646	0,8565	-0,2014	-0,4459	-2,31E-15
Crumb red index (a*)	0,7219	0,4847	-0,4668	-0,1613	1,73E-15
Crumb yellow index (b*)	0,8968	-0,2152	-0,3841	0,04334	-1,87E-14
Crust Brown index (100-L*)	0,3275	0,732	-0,5922	-0,07818	2,69E-15
Crust red index (a*)	-0,2011	-0,8949	0,09043	0,3881	3,21E-15
Crust yellow index (b*)	-0,4251	-0,8516	0,2837	0,1168	-3,44E-17
